# Rationale and design of a cohort study evaluating triage of acute chest pain in out-of-hours primary care in the Netherlands (TRACE)

**DOI:** 10.1017/S1463423620000122

**Published:** 2020-05-08

**Authors:** Amy Manten, Cuny J.J. Cuijpers, Remco Rietveld, Emma Groot, Freek van de Graaf, Sandra Voerman, Jelle C.L. Himmelreich, Wim A.M. Lucassen, Henk C.P.M. van Weert, Ralf E. Harskamp

**Affiliations:** 1 Department of General Practice, Amsterdam UMC, University of Amsterdam, Amsterdam Public Health, Academic Medical Center, Meibergdreef 9, AZ, Amsterdam, The Netherlands; 2 Huisartsenorganisatie Noord-Kennemerland, Hertog Aalbrechtweg 5a, Alkmaar, The Netherlands

**Keywords:** chest pain, primary care, triage, acute coronary syndrome

## Abstract

The aims of this study are (1) to evaluate the performance of current triage for chest pain; (2) to describe the case mix of patients undergoing triage for chest pain; and (3) to identify opportunities to improve performance of current Dutch triage system for chest pain. Chest pain is a common symptom, and identifying patients with chest pain that require urgent care can be quite challenging. Making the correct assessment is even harder during telephone triage. Temporal trends show that the referral threshold has lowered over time, resulting in overcrowding of first responders and emergency services. While various stakeholders advocate for a more efficient triage system, careful evaluation of the performance of the current triage in primary care is lacking. TRiage of Acute Chest pain Evaluation in primary care (TRACE) is a large cohort study designed to describe the current Dutch triage system for chest pain and subsequently evaluate triage performance in regard to clinical outcomes. The study consists of consecutive patients who contacted the out-of-hours primary care facility with chest pain in the region of Alkmaar, the Netherlands, in 2017, with follow-up for clinical outcomes out to August 2019. The primary outcome of interest is ‘major event’, which is defined as the occurrence of death from any cause, acute coronary syndrome, urgent coronary revascularization, or other high-risk diagnoses in which delay is inadmissible and hospitalization is necessary. We will evaluate the performance of the triage system by assessing the ability of the triage system to correctly classify patients regarding urgency (accuracy), the proportion of safe actions following triage (safety) as well as rightfully deployed ambulances (efficacy). TRACE is designed to describe the current Dutch triage system for chest pain in primary care and to subsequently evaluate triage performance in regard to clinical outcomes.

## Background and rationale

**Figure 1.**
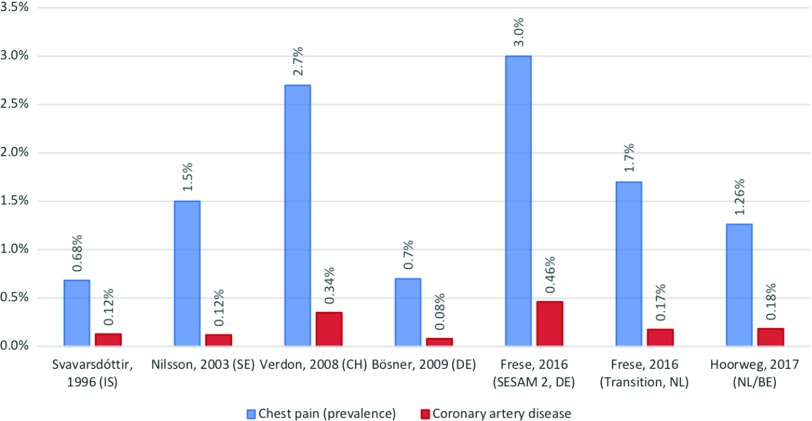
The prevalence of chest pain and the occurrence of coronary artery disease.

Chest pain is a common symptom with a lifetime prevalence of 20–40%, and in primary care, about 1 in every 50 consultations is related to chest pain (Svavarsdóttir *et al.*, 1996; Nilsson *et al.*, 2003; Ruigomez *et al.*, 2006; Verdon *et al.*, 2008; Bösner *et al.*, 2009; Bösner *et al.*, 2010; McConaghy and Oza, 2013; Frese *et al.*, 2016; Aerts *et al.*, 2017; Hoorweg *et al.*, 2017; Harskamp *et al.*, 2018). A minority of these cases have an underlying heart condition, with a pre-consultation probability that varies from 1.5% to10% (Figure 1) (Svavarsdóttir *et al.*, 1996; Nilsson *et al.*, 2003; Verdon *et al.*, 2008; Bösner *et al.*, 2010; McConaghy and Oza, 2013; Haasenritter *et al.*, 2015; Leite *et al.*, 2015; Aerts *et al.*, 2017; Hoorweg *et al.*, 2017; Harskamp *et al.*, 2018). Early detection of these disease states is warranted as adequate treatment prevents complications, such as heart failure, ventricular arrhythmias, as well as death itself (Deakin, 2006; van Ierland *et al.*, 2011). Unfortunately symptoms may not always be as straightforward, and this can be particularly challenging for out-of-hours primary care facilities where initial triage occurs mainly over the telephone. Triage protocols have therefore been put in place in order to adequately and quickly identify urgent cases, while simultaneously preventing iatrogenic harm by exposure to unnecessary ambulance activation, hospital admissions, diagnostic evaluation, and/or treatment in non-urgent cases (Bösner *et al.*, 2009; Bösner *et al.*, 2010; Frese *et al.*, 2016). The inability to perform physical examination and the absence of clinical risk scores typically used in clinical settings complicates telephone triage even further.

The blue charts show the prevalence of chest pain among patients in primary care for each study. The occurrence of coronary artery disease among patients presenting with chest pain is illustrated by the red charts.

The health care system in the Netherlands is designed to provide equal access to all citizens. Every Dutch citizen is enlisted with a local primary care physician’s (PCP) office, which keeps an electronic patient record file with all health care information (including correspondence from other health care facilities) for each of its patients. PCPs across the country are organized into regional, large-scale PCP cooperatives. The responsibilities of these cooperatives include the after-hours acute primary care services of the affiliated PCPs in that region through out-of-hours care facilities (Smits *et al.*, 2017). Most out-of-hours care facilities use the Dutch triage guideline [Nederlandse Triage Standaard (NTS)] (Domus Medica, 2014). The NTS for chest pain (‘pijn thorax’) is derived from extrapolating hospital triage questions and consensus of expert opinion. These triage questions have never been cross-validated with clinical outcomes in a primary care population with undifferentiated chest pain (van Ierland *et al.*, 2011). Additionally, we have poor understanding of the initial presentation of patients with chest pain in out-of-hours primary care, as prior research has exclusively focused on either office hours primary care or emergency departments (McConaghy and Oza, 2013; Frese *et al.*, 2016; Hoorweg *et al.*, 2017). As a result, the diagnostic value of the NTS in out-of-hours primary care has been largely criticized by PCPs as well as ambulance services (Keizer *et al.*, 2014). Triage inefficiency has been named as a main factor leading to frequent consultations for non-urgent cases and a high rate of unnecessary ambulance deployments, but other factors also play(ed) a role (i.e., organizational and cultural changes, financial and legal incentives) (Keizer *et al.*, 2014; Smits *et al.*, 2017; Smits and Verheij, 2017). Additionally, prior research has concluded that the urgency is underestimated in 19% of cases, potentially leading to unsafe situations (Giesen *et al.*, 2007; Keizer *et al.*, 2014; Ambulancezorg Nederland, 2017; Smits *et al.*, 2017; Smits and Verheij, 2017; Zeilstra and Giesen, 2017).

As such, we decided to conduct the TRiage of Acute Chest pain Evaluation (TRACE in primary care) study that designed to validate and optimize current triage decision-making in primary care by evaluating the current triage of chest pain in relationship to predicting major event. The first objective is to evaluate the performance of the current triage protocol/system for chest pain. The second objective is to describe symptom presentation, patient characteristics, triage decisions, final diagnoses, and clinical outcomes in patients presenting with chest pain in out-of-hours primary care. Finally, we explore opportunities to further improve triage safety and efficacy.

## Methods

The TRACE study involves a retrospective, observational cohort of consecutive patients who contacted a Dutch primary care out-of-hours facility with symptoms of chest pain. We reported our study protocol using the ‘Strengthening the reporting of observational studies in epidemiology’ checklist (von Elm *et al.*, 2007).

### Study population

**Figure 2.**
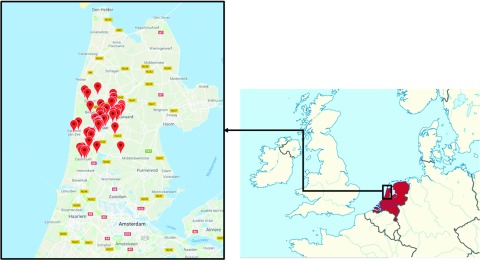
Regional coverage of the HONK network.

TRACE involves a partnership with Huisartsenorganisatie Noord-Kennermerland (HONK). The HONK organization is the primary care cooperative of the surrounding region of Alkmaar, the Netherlands (Figure 2), comprising approximately 240 000 individuals. In the Alkmaar region, the majority of the inhabitants are of Dutch or European ancestry, with 12.8% being of non-Western/non-Caucasian descent. Of those, the largest minorities are Turkish (2.9%), Moroccan (2.0%), Surinamese (1.5%), and Antillean (1.2) (Centraal Bureau voor de Statistiek, 2013). The out-of-hours primary care facility in Alkmaar is responsible for the after-hours primary care services to patients of the 98 affiliated primary care day practices. In the Netherlands, all inhabitants are registered at one primary care practice in their area of residence. TRACE will include all patients who telephonically and/or physically contacted the out-of-hours primary care facility in Alkmaar with a complaint of chest pain between 1 January 2017 and 31 December 2017. Patients aged 18 years and over at the time of initial contact with a primary or secondary complaint of chest pain will be eligible for inclusion. For patients who reach out to the HONK with chest pain on multiple occasions during the inclusion window, only the first encounter will be included.

The regional coverage of the HONK network in the province of Noord-Holland, north of Amsterdam. Each pin on the map represents an affiliated primary care practice.

**Figure 3.**
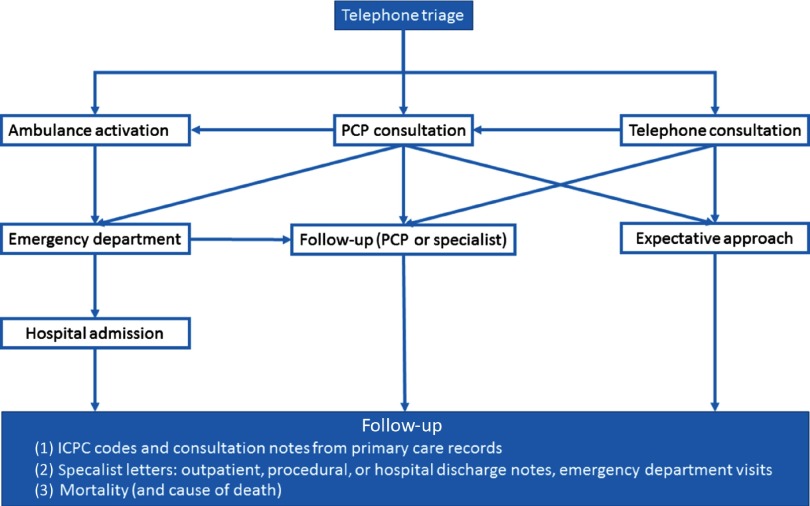
Flowchart of telephone triage and actions after triage.

This figure depicts the actions that can be taken after telephone triage of chest pain.

### Inclusion procedure

All patients who contacted the HONK with chest pain in 2017 will receive information regarding the TRACE study by mail. The envelope includes an information leaflet and a form with the request to withdraw from participation (‘opt out’). Data of patients who opt-out will not be used in any form for this study. Moreover, we will also not collect data on patients with unstable vital signs (U0 urgency), or when follow-up data cannot be collected (e.g., individuals who were not administered with a PCP at the time of initial contact, such as visitors/tourists, asylum seekers). All PCPs affiliated with the HONK will also be informed of the conduct of the study. We will also telephone PCP offices to schedule an appointment to complete follow-up information and to obtain current vital status information.

### Triage system used at the out-of-hours facility

To assure nationwide consistency, primary care cooperatives use a standardized, software-based triage system, which relies on algorithms developed, maintained, and exploited by the NTS (Domus Medica, 2014). The out-of-hours facilities are directly accessible for patients, but the preferred route of contact is by telephone. During a telephone contact, initial triage is carried out by trained triage nurses and supervised by on-site PCPs (Smits *et al.*, 2017). Triage nurses start by ensuring the hemodynamic status of a patient, and when unstable vital signs are present, the highest urgency level is activated, resulting in immediate ambulance deployment. In the vast majority of patients, these vital signs are stable and triage for chest pain follows, and questions include chest pain characteristics, that is, the type of pain, duration, severity, course, localization, and whether the patient experienced radiation of the pain or symptoms of sweating, nausea, or vomiting. Additionally, triage nurses can explore the medical history, medication use or the presence of accompanying symptoms, such as dyspnea or dizziness. Based on the answers to the standardized questions, an algorithm will generate an urgency category varying from urgency level 0 to 5 (U0–U5) as shown in Table 1. Possible actions following telephone triage include ambulance deployment, consultation by a PCP (at the center or a home visit) or telephone advice, as shown in Figure 3. The urgency classification determines the course of action and its time frame (see Table 1). When triage nurses suspect that a different urgency level would be more fitting to the patients’ symptom, they can overrule the suggested urgency after consulting the attending physician.

**Table 1.** Urgency classification according to NTS

Urgency codes	Definitions	In time
U0	No vital signs	Immediate – resuscitation
U1	Life threatening	As soon as possible – ambulance
U2	Emergent	Care <60 min
U3	Urgent	Care within several hours
U4	Non-urgent	Care within 24 h
U5	Advice	Next workday – advice

NTS = Nederlandse Triage Standaard.

### Data collection

Data collection for TRACE will consist of two parts: the first part involves collection of baseline data and the second involves collection of follow-up and clinical outcomes data. Collection of follow-up data will be performed once data entry for baseline characteristics has been completed. We will enter all data into Castor Electronic Data Capture (Castor EDC) (Ciwit, 2016), a cloud-based electronic data capturing platform in which the data of each participant can be registered within an electronic case report form (eCRF). Castor EDC includes an audit trail. We will pseudonymize all patient data before data entry.

#### Baseline data

We will regard the information available at the time of the index (telephone) consultation of the out-of-hours facility as baseline data. This information will consist of demographics, telephone triage outcome in terms of ascribed urgency, and the management that followed triage, as recorded by the HONK. We will counter inter-observer variability of entered data by developing agreements on data registering of each variable, as well as by an internal audit of data by three researchers (A.M., W.A.L., R.E.H.). For cross-validation purposes, we will also retrieve information from PCPs’ offices regarding baseline characteristics and medication use (at time of baseline consultation).

#### Follow-up data

For data on clinical outcomes, we will use information derived from electronic patient records, including correspondence regarding hospital visits and outpatient consultations. Relevant documentation, such as specialist letters regarding follow-up of chest pain, will be pseudonymized and uploaded into the eCRF. This follow-up information will include both final diagnosis in relation to the index consultation and other relevant outcomes during the entirety of the follow-up period. We will gather follow-up data of all patients up to 12–30 months after index telephone triage. Our research team will schedule appointments with the practice to allow our researchers to visit the practice to collect follow-up data. In small primary care practices with few participants, and limited work space, we will contact the PCP to discuss the method of data entry. In these practices, PCPs will either allow one of the researchers to visit the practice for data collection, or the PCP will receive a personal token to log in to our eCRF and process the information of these patients themselves. We believe that via this approach we are sensitive toward the logistical challenges (i.e., providing a work station) that our study may impose on small practices. Still, as our team provides flexibility in our data collection visits, we anticipate that the vast majority will opt for our research team to collect follow-up information. Collection of follow-up data will be concluded in August 2019.

### Final diagnosis: major and non-major events

We will evaluate triage performance by comparing the initial triage management at the HONK with the final diagnosis and clinical outcomes as retrieved from the patient records at the PCPs’ offices. In order to do so, we will establish the final diagnosis for each patient and evaluate whether this diagnosis should be identified as a ‘major event’. Subsequently, we will review the necessity of ambulance activation in hindsight when regarding the final diagnosis.

First, we will determine final diagnosis. In primary care, diagnoses are registered as International Classification of Primary Care (ICPC) codes. We will use the ICPC codes that are registered in the patient records at the time of clinical follow-up. We anticipate to encounter a number of cases in which the registered ICPC code does not match with final diagnosis based on information from electronic health records. In case of doubt about the validity of the registered ICPC code, the necessity of revision of the ICPC code based on the full patient file (including information on diagnostic work-up and correspondence by specialists) will be decided on by discussion among the research group and, in case of disagreement, by an independent expert who is blinded for initial management. The (misclassified) ICPC code will be adjusted to the more fitting code in the eCRF according to this final judgment. Such changes will be recorded in data logs.

Second, final diagnoses will be classified as either ‘major’ or ‘non-major’ type events. This distinction, as shown in Table 2, will be based on whether patients suffered from an acute coronary syndrome (ACS), a major adverse cardiovascular event (MACE), or other urgent clinical outcome. We defined MACE as the occurrence of all mortality, myocardial infarction/unstable angina, or coronary revascularization, as used in the HEART score (Van Den Berg and Body, 2017). Data on mortality are obtained from various sources, including the HONK database (linkage) and from electronic health records from the daytime PCPs. The occurrence of ACS and coronary revascularization is based on source documentation, based on a specialist and/or hospital discharge letter. Clinical outcomes other than ACS or MACE will be classified as major events when they fulfill all of the following three criteria: (1) ambulance activation was justifiable in hindsight, (2) the patient was hospitalized, and (3) final diagnosis can be linked to the initial complaint of chest pain. Major events are separated further into events occurring within 6 weeks and events occurring later than 6 weeks following initial contact. This division is based on the assumption that most major events associated with initial contact would manifest within a period of 6 weeks. Major event occurring later than 6 weeks for instance include patients who suffered from cardiac ischemia later on or participants who died within the follow-up period. We decided to include a longer follow-up period as it may counter some of the effects of verification bias, as patients initially not referred to a specialist, may have been referred/diagnosed at a later stage.

**Table 2.** Distribution of major and non-major events

	Final diagnosis	Management
Major event	ACS	
	MACE	
	Pulmonary embolism	
	Thoracic aortic aneurysm	
	Severe/acute congestive heart failure	Hospitalization
	Severe peri(myo)carditis	Hospitalization
	(Tension) pneumothorax	Hospitalization/drain
	Severe pneumonia	Hospitalization
	Symptomatic atrial fibrillation	First onset/hospitalization/ECV or converted through medication.
	Aortic valve stenosis	Symptomatic
	Inflammatory processes such as appendicitis, pancreatitis, cholecystitis	Hospitalization
	Other, such as exacerbation of COPD or hypertensive crisis	
Non-major event	Mild congestive heart failure	Outpatient treatment
	Mild peri(myo)carditis	Outpatient treatment
	Mild pneumothorax	Outpatient treatment
	Mild pneumonia	Outpatient treatment
	Atrial fibrillation	Recurrence/paroxysmal
	Pericarditis	Outpatient treatment
	Hypertension	Outpatient treatment
	Angina pectoris	Stable, outpatient treatment
	Skeletomuscular	
	Traumatic	
	Gastric/esophagus problems	
	Mild respiratory problems (such as viral infections)	
	Mental health/panic attack/anxiety disorder	

ACS = acute coronary syndrome; MACE = major adverse cardiovascular event; ECV = Electrical Cardioversion; COPD = Chronic obstructive pulmonary disease.

### Triage performance

We will assess the performance of the current triage system by its ‘accuracy’, ‘safety’, and ‘efficacy’ regarding the ability to differentiate major from non-major events. We will define ‘accuracy’ as the overall probability that a patient is correctly classified by the triage system regarding the urgency of the final diagnosis. We will define correct classification as ambulance activation at initial consultation for a participant with a major event and no ambulance activation for a participant with a non-major event. We will determine ‘safety’ as one minus the false negative rate (i.e., the proportion of cases falsely assessed as non-major divided by the total number of included triage cases). The triage system’s safety therefore ranges from 0 to 1, where a safety close or equal to 1 indicates a low proportion of cases with a ‘missed’ major event. ‘Efficacy’ refers to the proportion of ambulance deployments that involved a major event (true positives) divided by the total number of ambulance activations.

Additionally, we will also compare the urgency levels overruled by triage nurse (and PCP) versus not overruled and what effect this overruling has on safety, efficacy, and accuracy. The action of overruling of the triage nurse is documented in the HONK database.

### Statistical analysis

We will perform descriptive analyses on symptom presentation, patient characteristics, triage decisions, final diagnosis, and clinical outcomes. We will display discrete variables as number and percentages and continuous variables as means ± standard deviations or median and interquartile range for non-Gaussian distributions. We will compare continuous variables using Student’s *t*-test and proportions using the Fisher’s exact test or Pearson’s chi-square test. We will use two-tailed tests.

We will evaluate the current NTS triage performance using safety, efficacy, and accuracy regarding major events and ambulance activity. We will perform a stratified analysis of triage performance comparing the sole use of (computer/algorithm-based) NTS telephone triage recommendation or the actual every-day use which integrates overruling of the triage nurse or physician.

We will evaluate diagnostic accuracy of the triage questions by their sensitivity, specificity, and predictive values regarding the outcomes (1) major event and (2) MACE. We will also assess discrimination (C-statistics) and calibration (assessing calibration plots, and performing modified Hosmer–Lemeshow tests) of triage questions. We anticipate to include 1500–2000 patients with chest pain over a 1-year observation period based on prior the site’s telephone consultation history. Of those, we estimate that approximately 10% of cases will involve a major event that requires immediate referral. Based on the rule of thumb that one variable would require at least 10 cases of the primary outcome, this would provide us with sufficient power to allow for the analysis of 15–20 variables.

We will conduct all analyses using IBM SPSS Statistics for Windows, Version 25 (IBM Corp., Released 2017, Armonk, NY, USA). We will evaluate statistical significance in all analyses at the 0.05 level.

### Ethical approval

The study proposal of TRACE was assessed by the Medical Ethical Committee (METC) (institutional review board) of the Amsterdam University Medical Centers, location Academic Medical Center (AMC). Given the observational design of the study, the METC exempted TRACE for full evaluation, as provided in writing on 13 February 2018 (reference number W18_035#18.053). All patients who sought telephone contact with chest pain in the year 2017 will receive a letter from the HONK with information about the TRACE study. They will also be provided with the possibility to withdraw from the study cohort through an attached ‘opt-out-form’ and return envelope, in case of objection. These actions have been reviewed by internal legal consultation and data protection representatives, and are (for the current study) within the boundaries of the new General Data Protection Regulation installed in May 2018 and the Dutch law on Medical Research in Humans. A summary of the study protocol has been published on Netherland trial register of the Dutch Cochrane group (Trial NL7581, https://www.trialregister.nl/trial/7581).

## Discussion

Chest pain provides a challenge for telephone triage, particularly for atypical symptoms and/or supposedly low-risk patients. This study will provide new insights as we will apply the actual clinical outcome as the reference, instead of ‘expert opinion’ which is the current basis of the NTS triage system. Critical evaluation of our current triage system using clinical follow-up data may provide us with the opportunity to further refine triage in order to maximize safety and efficacy. The available data would allow us to identify additional predictors for adverse clinical outcomes that could be taken into account when developing future updates of the current NTS triage system. While the Dutch health care system has unique features, the findings of TRACE will in general still be applicable in other countries with a strong primary care presence in acute/urgent care.

### Prior and ongoing studies

A comparable study evaluating the optimization of telephone triage is currently conducted by Erkelens *et al.* (2019) in the region of Utrecht, the Netherlands. This study uses the telephone triage audio recordings of patients with symptoms suggestive of acute cardiovascular disease in out-of-hours primary care. Instead of initial management, Erkelens *et al.* focus on the diagnostic accuracy of the allocated urgency level after telephone triage. The results of this study are likely to complement TRACE in attaining a complete picture of current triage standards in the Netherlands.

### Strengths and limitations

The TRACE study reflects real-world care of a contemporary cohort, with a patient population that is reflective of a Western European population. Use of primary care and affiliated resources in the Alkmaar region is comparable with the Dutch average, as is the use of medications and chronic medical conditions (https://www.waarstaatjegemeente.nl/dashboard/Zorggebruik/). Moreover, since consecutive patients will be enrolled over the course of a year, we minimize possible selection or seasonal bias. Another strength is the robust follow-up and documentation of both cardiac life-threatening diagnoses (ACS/MACE) and other significant clinical outcomes. Furthermore, the urgency of the final diagnosis will be evaluated manually for each participant instead of relying solely on ICPC code registering. A limitation of our study is verification bias. We will only have follow-up documentation available of patients who were sent to secondary care for further evaluation. In order words, we only have partial verification, where only those triaged to secondary care (either immediately due to telephone triage or indirectly after PCP evaluation) will receive complete evaluation for rule out of major events. The magnitude of verification bias depends on the number of cases referred. We try to minimize this effect by having an extended follow-up, to allow for delayed-type/missed events to be captured. The use of extended follow-up using routine medical care data also introduces information bias, of which loss to follow-up is one of importance. Another form of bias that may play is confusion bias. With this form of bias, other (temporal) factors that are related to the ultimate outcome (i.e., age or sex), but not related to (or known at time of) triage, can modulate the effects of triage. Also, we run the potential for data manipulation errors, as the follow-up data of some practices (with <20 participants) will be entered by the PCPs themselves, instead of by our research team. Lastly, while the findings of our study may be reflective of a Dutch urgent primary care setting, it is uncertain how these findings extrapolate to countries with health care systems where primary care plays a less prominent role in urgent care.

## Conclusion

TRACE is designed to evaluate telephone triage of patients presenting with chest pain in out-of-hours primary care. The study will focus on various aspects of telephone triage, including symptom presentation, triage decision-making, and triage performance. The main goal will be to provide insight in our current triage decisions and to create recommendations to enhance the safety and efficacy.
